# Social Skills in Preschool Children from Teachers’ Perspectives

**DOI:** 10.3390/children6050064

**Published:** 2019-05-01

**Authors:** Maryam Maleki, Minoo Mitra Chehrzad, Ehsan Kazemnezhad Leyli, Abbas Mardani, Mojtaba Vaismoradi

**Affiliations:** 1 School of Nursing and Midwifery, Shahroud University of Medical Sciences, Shahroud 3614773955, Iran; malekimaryamn92@gmail.com; 2 School of Nursing and Midwifery, Guilan University of Medical Sciences, Guilan 4199613776, Iran; chehrzad@gums.ac.ir (M.M.C.); eh_kazem@gums.ac.ir (E.K.L.); 3 Faculty of Nursing and Health Sciences, Nord University, 8049 Bodø, Norway; mojtaba.vaismoradi@nord.no

**Keywords:** Social skill, preschool children, teachers

## Abstract

Preschool is one of the most important periods in a child’s life, and it can influence their social development. A lack of attention to this important life period can increase the risk of serious injuries to a child’s growth and development. The aim of this study was to examine the level of social skills in preschool children from teachers’ perspectives and investigate the relationship between social skills and the child’s environmental and cultural background. A cross-sectional study was conducted on 546 children studying in the preschool centers of Rasht city, Iran. They were selected using a multistage cluster sampling method. Data was gathered using the children’s and teachers’ demographic questionnaire and the Social Skills Rating System-Teachers (SSRS-T). Descriptive and inferential statistics were used for data analysis via SPSS software. It was found that the majority of children had moderate skills in terms of cooperation, assertion, self-control, and total social skills. Also, a statistically significant association was reported between total social skill scores and the mother’s age, mother’s education level, mother’s job, family’s income, teacher teaching experiences, and number of children in each classroom. The Iranian children were at a relatively low risk of problems with social skills. To improve children’s social skills, more attention should be paid to factors related to familial and socioeconomic status such as income, parents’ education level, maternal age, teacher’s selection for this age group, and number of children in each classroom.

## 1. Introduction

Children are considered the national wealth of each society [1]. Therefore, sufficient attention should be paid to their physical, psychological, and social health, as well as growth and development [2,3]. The World Health Organization (WHO) has developed the life skills approach, which is situated in three main areas of social, cognitive, and coping-emotional skills [4]. Since individuals configure their life through social skills, they cannot live without them. Social skills have been defined as the complex set of skills and behaviors that empower an individual to interact appropriately with others and avoid unaccepted responses in the society [5].

According Elliott and Gresham, social skills can be measured by assessing children’s cooperation, assertion, and self-control [6]. Cooperation is a social interaction by which individuals develop their activities in an organized way and interact with each other to achieve a shared goal. It includes behaviors such as provision of help to others, partnership, and following rules and commands [7]. Assertion involves initiating behaviors such as obtaining information from others and introducing themselves to them [6]. Self-control is the expression of personal emotions and consists of behaviors in various situations that require appropriate reactions by the child [8]. 

Preschool age is a crucial period for social and emotional development [9,10]. Preschool children acquire social skills for an effective performance, adaption, education, and improvement in quality of life [11,12]. At this age, children develop social and personal skills, acquire problem-solving skills, develop academically, and learn to express their desires and feelings [12]. Deficits in social skills in this period can lead to internalizing and externalizing behavioral disorders, poor academic performance, inappropriate reconciliation in interpersonal relationships, educational and cognitive impairments, isolation, and psychological issues later in life [4,12,13,14]. Development of social skills in this age period is influenced by the family income, parent education level [15], parent age [16], home and school environment, peer relationships, and sociocultural background [17]. Childhood problems can be attributed to a lack of attention to children’s needs during this sensitive life period [18].

Differences in the development of social skills between boys and girls during the preschool period have been shown [19]. For instance, in an Iranian sample, girls showed higher social skills and academic competence, while boys had higher behavioral problems [20]. Many studies have reported higher levels of social skills in girls compared to boys [20,21,22], although some studies have found no gender differences in social skills [23,24]. Therefore, the association between gender differences during early childhood in terms of social skills needs further exploration [25].

Children mostly spend their time in social environments such as preschool centers, which impact the development of their social skills [26]. In this regard, preschool teachers represent the children’s first nonfamily caregivers. Since the education of preschool teachers is needed to acquire appropriate knowledge of the developmental process of children’s psychology, these teachers have a more in-depth knowledge of children’s development in the preschool period [27]. Therefore, preschool teachers are the most important sources of information about the social and emotional behaviors of children in social environments [20,26] and can provide a reliable report of children’s development [27]. In addition, these teachers can monitor and compare children in terms of development in the same environment [28,29].

Children’s social skills are influenced by environmental and cultural factors [30]. In different cultures, various emphases are placed on communication and socialization skills [31]. However, the association between social skills and cultural factors have not been studied in non-Western societies including Iran [20,31]. In the pre-school period with the age range of 3–6 years [32], children in Iran enter kindergarten and preschool centers. Preschool education (preprimary education) is an optional one-year period in which children that have reached five years of age and have reached the age of six are prepared to enter elementary school [33]. 

This study was conducted with the aim of improving our knowledge of social skills in preschool children from teachers’ perspectives, understanding differences between girls and boys in terms of social skills, and describing the relationship between social skills and the environmental and cultural background. 

## 2. Materials and Methods

### 2.1. Design and Participants

A cross-sectional study was conducted on all preschool children studying in preschool centers in Rasht city, Iran in 2015–2016. Inclusion criteria for the recruitment of samples were age of six years (born between September 23, 2009 to September 23, 2010), residence of Rasht city, no known physical and mental health problems, living with both parents, at least 3 months passed from the acquaintance of the child with the teacher, and studying at one of the preschool centers under the supervision of Rasht Ministry of Education (regions 1 and 2) and the Welfare Organization. 

The sample size was estimated using the results of Sheikhzakaryaie et al.’s study on preschool age children in the city of Tehran [30]. In addition, the standard deviation of the total scale in preschool children was 19 with a confidence level of 95%, assuming that the accuracy of estimating the average score of social skills of children from teachers’ perspectives was at least 2 scores. The sample size using the following formula and the cluster model design (design effect = 1.5) was calculated as follows:

(1) $$n=\frac{z^{2}s^{2}}{d^{2}}\Rightarrow n=\frac{1.96^{2}*19^{2}}{2^{2}}=350\Rightarrow n=350*1.5=525$$

**Figure 1:**
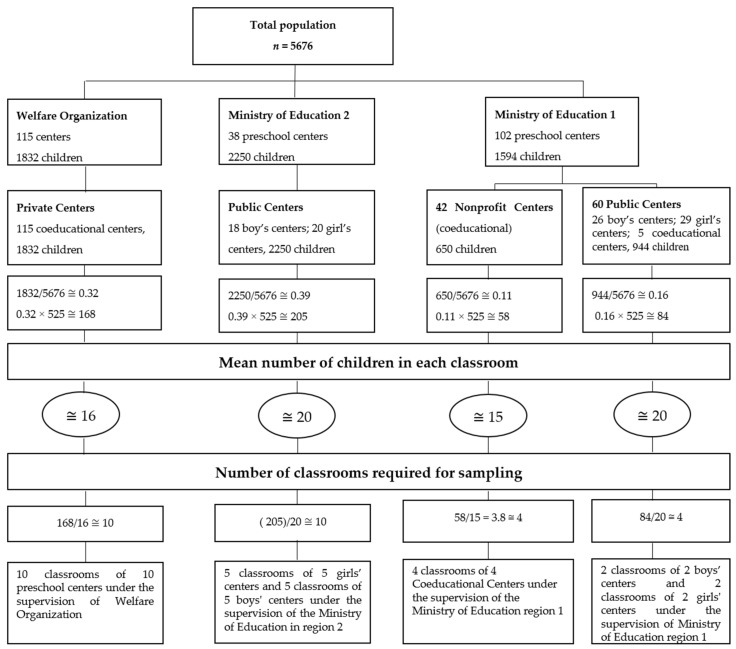
Process of sampling in this study.

A two-stage random cluster sampling method was used. The total number of preschool age children studying at preschool centers was obtained from education authorities. Next, the population was classified based on preschool centers under the supervision of the Ministry of Education region 1 (boy’s, girl’s, and coeducational preschool centers of the public and nonprofit types), the Ministry of Education region 2 (boy’s and girl’s preschool centers of the public type), and the Welfare Organization (coeducational preschool centers of the private type). The number of children in each of these classified preschool centers was divided into the total population of preschool children and then multiplied by the number of estimated samples (*n* = 525). The number of children who should be selected from public and nonprofit preschool centers under the supervision of Ministry of Education region 1 and 2, as well as private preschool centers under the supervision of the welfare organization, was calculated. The number was divided into the average number of children in each classroom, and the number of classrooms was calculated (*n* = 28 classes). The same number of preschool centers was selected randomly; then, from each center, only one classroom was randomly entered to the study (Figure 1).

### 2.2. Measures

Data was collected using the children’s and teachers’ demographic questionnaire and the Social Skills Rating System-Teachers (SSRS-T). The children’s demographic questionnaire included three categories of questions about children information (gender, birth rank, going to kindergarten), children familial information (parent age, parent education level, parent job, number of children in the family, and family income), and exclusion criteria (physical and mental health problems, living with both parents). The teachers’ demographic questionnaire included questions about age and teaching experience, as well as characteristics of the classroom (number of children in each classroom) and preschool centers (type of organizational affiliation, preschool type).

The Social Skills Rating System (SSRS), as the most comprehensive rating scale for the assessment of social skills, was used for data collection [2]. The SSRS-T assesses social skills from teacher’s perspectives. The psychometric properties of the SSRS-T have been reported in a previous study. Internal consistency using the calculation of the Cronbach’s alpha coefficient was 0.94. Moreover, the four-week test–retest reliability coefficient for social skills was 0.85 [34]. In Iran, the test–retest reliability within a 4–5-week interval was 71%, and the correlation coefficients between different subscales of social skills ranged from 38% to 66% [21]. 

The SSRS-T is a standardized 30-question questionnaire with a 3-point Likert scale (0 = Never, 1 = Sometimes, and 2 = Very Often). The SSRS-T consists of three subscales of cooperation (19 questions), assertion (8 questions), and self-control (3 questions), and the total social skills’ score is calculated by summing up all subscale scores. The score for the cooperation subscale is from 0 to 38, 0–16 for the assertion subscale, and 0–6 for the self-control subscale. The total social skills score ranges from 0 to 60. If the raw score calculated in each subscale and the total social skill are one standard deviation higher and lower than the mean, it indicates moderate social skills. If they are one standard deviation above the mean, it indicates high social skills, and if they are one standard deviation below the mean, it indicates low social skills [21,34]. In the normative sample, the percentages of children in the low, moderate, and high categories have been reported to be 16%, 68%, and 16%, respectively, for the teacher rating [35].

### 2.3. Data Collection

A total of 28 preschool centers were recruited randomly (Figure 1). Only one classroom in each preschool center was selected. In preschool centers with several preschool classrooms, a classroom was selected randomly. The data collection was conducted from December to January of the school year of 2015–2016. After obtaining permissions, children’s names were coded, and the questionnaires including the teachers’ demographic data and the SSRS-T questionnaires were delivered to the teachers to be completed. In addition, the children’s demographic questionnaire was completed by the children’s parents when they were referred to the preschool center. Considering the possibility of sample dropouts, 598 questionnaires were distributed for collecting data.

### 2.4. Ethical Consideration

The research proposal was approved by the Social Determinants of Health Research Center at Guilan University of Medical Sciences (decree code: IR.GUMS.REC.1394.52). The permission to enter the research zone was obtained prior to the study. The teachers and children’s parents were informed of the purpose and method of the study, and that collected data would remain confidential. The willing teachers and parents signed the informed consent form to enter the study.

### 2.5. Data Analysis

For data analysis, descriptive and inferential statistics via the SPSS v. 22 software (IBM, Armonk, NY, USA) were used. Raw data was entered into the software, and social skills’ scores were calculated for cooperation, assertion, self-control, and the total social skill. Data was evaluated in terms of normal distribution using the Shapiro–Wilk test and the Kolmogorov–Smirnov test. The one-way ANOVA test and independent samples t-test were used to examine the relationship between total social skills and research variables. In addition, the normative sample was used to divide the samples into three categories of low, moderate and high social skills.

## 3. Results

Out of 598 questionnaires, 52 questionnaires were excluded due to incomplete completion by the subjects (*n* = 17) and no compliance with exclusion criteria (*n* = 35). Therefore, 546 questionnaires were incorporated in the data analysis and reporting.

The demographic characteristics of the subjects are presented in Table 1. The majority of them were boys (57.9%), single children (52.2%), and the first child in the family (65.6%). In addition, 45.6% of them went to kindergarten during childhood. The majority of the children’s mothers (57.3%) and fathers (57.3%) were 30–40 years old. The mothers had a high school education level (46.1%) and were housekeepers (74.4%), but the fathers had an academic education level (38.7%) and were self-employed (60%). Most of the children had families with an income of $150–300 per month (49.6%). The teachers were 35.36 ± 5.29 years old with a teaching experience of 8.86 ± 3.85 years. The children were studying at public preschools (57.5%) and were in classrooms with 11–20 students in each classroom (42.7%).

A statistically significant association was found between the children’s total scores of social skills and the mother’s age (*p* = 0.04). According to the post hoc test, a statistically significant difference was found between the children with mothers aged <30 years and the children with mothers aged between 30–40 years (mean difference (MD) = −2.25, *p* = 0.01).

In addition, there was a statically significant difference between the mother’s education level and the children’s total scores of social skills (*p* = 0.01), and based on the post hoc test, there were statistically significant differences between the children with mothers with the academic education level and the children with the elementary education level (MD = 4.44, *p* = 0.04), and the children with mothers with the guidance school education level (MD = 3.39, *p* = 0.01), as well as the children with mothers with a high school education level (MD = 2.13, *p* = 0.01). In addition, those children whose mothers were employees achieved higher scores for their social skills (*p* = 0.04). A statistically significant difference was reported between the social skills’ scores and the children’s family income (*p* < 0.001). According to the post hoc test, a statistically significant difference was found between the children with a family income of $150–300 and the children with the family income >$300 (MD = −3.65, *p* < 0.001). In addition, a statistically significant difference between the teachers’ teaching experiences and the social skills’ scores was observed (*p* = 0.006), and the post hoc test revealed a statistically significant difference between the children with teachers with less than 5 years of teaching experience and the children with teachers having 5–10 years of teaching experience (MD = −3.62, *p* = 0.001). The same result was found in children with teachers with 5–10 years of teaching experience and the children with teachers with more than 10 years of teaching experience (MD = 1.94, *p* = 0.04). Finally, there was a statistically significant difference between the number of children in each classroom and the social skills’ scores (*p* < 0.001). According to the post hoc test, a statistically significant difference was found between those children who were studying in classrooms with more than 30 students and those children who were in classrooms with 11–20 students (MD = −4.17, *p* < 0.001), and the children in classrooms with 21–30 students (MD = −4.07, *p* < 0.001) (Table 1).

In addition, from the teachers ‘perspectives, the majority of the children had moderate skills in terms of cooperation (65%), assertion (67.2%), self-control (72.3%), and the total scores of social skills (67.4%) (Table 2).

## 4. Discussion

This study aimed to investigate social skills in preschool children from teachers’ perspectives and to investigate the relationship between social skills and the environmental and cultural background. Social skills enable children to develop positive relationships with teachers and peers that foster their success at school [10]. According to the findings of this study, the social skills of most children in the subscales of cooperation, assertion, self-control, and total social skills were at the moderate level. The research by Sheikhzakariae (2012) on the social skills of preschool children in the public centers of Tehran showed that the social skills of preschool children from teachers’ perspectives were at the moderate level in all subscales of social skills and total social skills [36]. In addition, Tan and Camras reported that the social skills of children were at the moderate level [35]. The children in the preschool centers acquired their first experience of socialization, and their initial experiences were at the moderate level from the teachers’ perspectives. However, Garmaroudi and Vahedi Nia showed that the social skills of middle and high school students were at the low level [37]. The reason for differences could be variations in the personal characteristics of each age group [38].

Most studies have shown that girls’ social skills are higher than those of boys. Conversely, in the present study, no difference between the total social skills of girls and boys was reported, which could be attributed to the Iranian context and related societal expectations. Gender roles and behaviors are educated by cultural stereotypes [20]. In the Iranian context, female children are expected to be more cooperative, submissive to tasks, kind, gentle, responsive, and empathic than boys in childhood [31]. Therefore, prescribing more other-oriented and well-controlled behaviors for girls than for boys are expected [20]. Since Iranian teachers commonly show less tolerance for girls’ social problems, they evaluate girls’ social skills in a lower level [21]. Taverna et al. believes that cultural differences have very important roles in creating different expectations in children [39].

The finding of this study showed a significant association between the children’s social skill scores and a high family income and high maternal education level. Higher parental education levels enable parents to acquire more knowledge and skills about child development [15]. In addition, it is believed that higher maternal education levels compared to those in fathers are associated with positive parental attitudes such as talking to children warmly or supportively, which is associated with better child development outcomes including social skills [15,40]. Furthermore, high family incomes enable them to invest more in their children’s growth and development in terms of goods and services, stimulation of learning, living standards, and living environment that fosters the child’s development [41,42]. On the other hand, Berry and O’Connor stated that poverty is a predictor of poor social skills in preschool children [43]. Conversely, Kumari and Khadi reported no relationship between children’s social skills and family income status [44].

The results of this study showed a significant association between the scores of social skills and the mother’s age. It is believed that older maternal age is associated with positive parental behaviors, including the mother–child bond, which can influence a child’s social development [45,46]. Younger mothers may have less parental skills [46,47], because mental health and maturity are enhanced when the mother grows older [16].

It was found that the children with employed mothers achieved higher scores of social skills. Maternal employment may have negative effects on maternal tasks, but early maternal employment is associated with both positive and negative child outcomes [48,49]. For instance, maternal employment can lead to an early-starting and long-lasting care by others and an earlier entrance into the social world. In the preschool period, it helps with the development of social skills and adaptation, and also leads to a reduction in the risk of psychological problems and isolation [48,50]. In addition, employment brings more financial resources into home and allows families to afford high-quality child care and services [50,51].

The findings of this study showed that children with teachers having 5–10 years’ work experience achieved higher scores in social skills. Preschool education is a critical period for the development of social skills [52]. Parents and teachers influence the development of children, but the teacher has an important role in forming children’s perspectives regarding school and their work habits [53]. According to Sheikhzakariae, the social skills of preschool children who had older teachers and with more than 10 years’ work experience were higher [36]. From a social-cognitive perspective, those teachers who have better teacher–child interactions can educate children how to adapt to and solve social problems and teach social and cognitive skills, which can improve the development of social skills [26,54]. Therefore, teachers with more work experience have higher skills in the education of social skills and are appropriate role models for children [26,55].

The number of students in the classroom was one of the indicators of social skills’ development. Students in smaller classrooms in their early years of schools have a better performance. In addition, classrooms with less number of students enable teachers to perform their teaching tasks more effectively [56]. Fredriksson et al. showed that children with a lower number of children in the classroom in their early school years benefited more from the education given by the teacher [57]. Therefore, classrooms with more than 30 children reduces the level of social interactions between the child and the teacher, which affects children’s social skills.

The present study was carried out on Iranian preschool children; therefore, the generalization of the results to other sociocultural contexts should be performed with caution. Furthermore, the social skills of children were evaluated from the teachers’ point of views. Therefore, parents’ perspectives and the use of other data collection methods, such as observations, are needed to contextualize the complexity of children’s social skills. In addition, samples were limited to preschool centers and children in their 6^th^ year of age, and other children were not included, which are factors that should be considered in future studies.

### Contributions and Implications

Since data regarding social skills was collected from teachers as the reliable sources of development data in the school setting, our findings extend previous knowledge of social skills in the preschool period. Overall, this study recognized the importance of family income, parental education, maternal age, maternal employment, teacher’s experience, and preschool classroom in terms of numbers of children on children’s social skills. Policymakers and programmers should educate teachers to stimulate the development of children’s social skills through group work and collaborations [26,58]. In addition, younger mothers should be educated about parental tasks to enhance their positive parental behaviors, which can lead to better children’s social development [59,60].

## 5. Conclusions

The majority of children had moderate skills in terms of cooperation, assertion, self-control, and total social skills. In addition, a statistically significant association was reported between total social skill scores and the mother’s age, mother’s education, mother’s job, family’s income, teacher’s teaching experience, and number of children in each classroom. The Iranian children were at a relatively low risk of problems with social skills. To improve children’s social skills, more attention should be paid to factors related to familial socioeconomic status such as the family income, parent education level, maternal age, teacher’s selection for this age group, and the number of children in each classroom.

## Figures and Tables

**Table 1:** The participants’ characteristics and their associations with the total scores of social skills of preschool children from the teachers’ perspectives.

Independent Variables		Children	Total Social Skill Scores	F ^#^	*p*-Value *
		*n* (%)	Mean (SD)		
Child’s gender	Girl	230 (42.1)	39.13 (9.60)	−0.360 ^##^	0.71 **
	Boy	316 (57.9)	39.43 (9.61)		
Number of children in the family	One	285 (52.2)	39.39 (9.36)	0.074	0.92
	Two	223 (40.8)	39.27 (10.16)		
	Third or more	38 (7)	38.76 (8.10)		
Child’s birth rank	First	358 (65.6)	39.44 (9.58)	0.123	0.88
	Second	166 (30.4)	39.09 (9.85)		
	Third or more	22 (4)	38.68 (8.24)		
Going to kindergarten	Yes	249 (45.6)	40.09 (9.65)	1.76 ^##^	0.07 **
	No	297 (54.4)	38.63 (9.52)		
Mother’s age	<30	173 (31.7)	37.82 (9.78)	**3.10**	**0.04**
	30–40	313 (57.3)	40.07 (9.30)		
	>40	60 (11)	39.58 (10.30)		
Father’s age	<30	56 (10.3)	37 (10.68)	2.54	0.08
	30–40	313 (57.3)	39.17 (9.41)		
	>40	177 (32.4)	40.26 (9.49)		
Mother’s education level	Elementary	21 (3.8)	36.42 (8.26)	**3.56**	**0.01**
	Guidance school	65 (11.9)	37.47 (9.17)		
	High school	251 (46.1)	38.73 (9.78)		
	Academic	208 (38.2)	40.86 (9.47)		
Father’s education level	Elementary	40 (7.3)	37.77 (10.34)	1.73	0.16
	Guidance school	102 (18.7)	38.08 (10.15)		
	High school	193 (35.3)	39.12 (9.17)		
	Academic	211 (38.7)	40.34 (9.52)		
Mother’s job	Employed	140 (25.6)	40.69 (9.58)	**1.98 ** ^**##**^	0.04 **
	Housekeeper	406 (74.4)	38.82 (9.57)		
Father’s job	Employee	210 (38.5)	39.64 (9.72)	1.86	0.15
	Self-employed	328 (60)	39.24 (9.49)		
	Unemployed	8 (1.5)	33 (9.97)		
Family’s income (USA dollar)	<150	67 (12.3)	38.98 (9.74)	**8.82**	**<0.001**
	150–300	271 (49.6)	37.76 (9.92)		
	>300	208 (38.1)	41.41 (8.75)		
Teacher age (year)	<30	76 (13.9)	39.86 (8.15)	2.07	0.12
	30–40	391 (71.6)	38.82 (9.68)		
	>40	79 (14.5)	41.13 (10.32)		
Teacher teaching experience (year)	<5	123 (22.5)	37.40 (8.66)	**5.23**	**0.006**
	5–10	168 (30.8)	41.02 (9.64)		
	>10	255 (46.7)	39.08 (9.84)		
Number of children in each classroom	≤10	31 (5.7)	38.93 (7.63)	**7.06**	**<0.001**
	20–11	233 (42.7)	40.64 (9.06)		
	30–21	122 (3.22)	40.54 (9.25)		
	≥30	160 (29.3)	36.47 (10.41)		
Type of organizational affiliation	Ministry of Education 1	202 (37)	38.97 (9.47)	0.201	0.81
	Ministry of Education 2	181 (33.1)	39.41 (10.16)		
	Welfare Organization	163 (23.9)	39.58 (9.20)		
Preschool type	Public	314 (57.5)	38.64 (9.84)	2.83	0.059
	Nonprofit	69 (12.6)	41.62 (9.13)		
	Private	163 (29.9)	39.58 (9.20)		

* One-way ANOVA; ** Independent Samples t-test; # F value; ## t-value.

**Table 2:** Social skills in the preschool age children from the teachers’ perspectives.

Dimensions of Social Skills	Total Mean (SD)	Range		
		Low	Moderate	High
Cooperation *n* (%)	25.44 (6.21)	93 (17)	355 (65)	98 (18)
Mean (SD)		15.66 (3.29)	25.69 (3.38)	33 (1.59)
Assertion *n* (%)	10.2 (3.50)	76 (14)	367 (67.2)	103 (18.8)
Mean (SD)		3.82 (2.02)	10.04 (1.91)	14.50 (0.71)
Self-control *n* (%)	3.84 (1.42)	93 (17)	395 (72.3)	58 (10.7)
Mean (SD)		1.50 (0.68)	4.07 (0.80)	6 (0.00)
Total social skills *n* (%)	39.30 (9.60)	84 (15.4)	368 (67.4)	94 (17.2)
Mean (SD)		23.70 (5.17)	39.56 (5.55)	52.21 (2.50)

## References

[1] Salmani-Barough N., Sharifi-Neiestanak N.D., Kazemnejad A., Pashaeypoor S. (2003). Self-concept and influential factors on it in the street children aged 6–12 years. Hayat.

[2] Vahedi S., Farrokhi F., Farajian F. (2012). Social competence and behavior problems in preschool children. Iran. J. Psychiatry.

[3] Jafari A. (2014). The effects of educational games on the social development of preschool children. Q. J. Educ. Psychol. Islam. Azad Uni. Tonekabon Branch.

[4] Karimi M., Keikhavani S., Mohammadi M. (2010). Efficacy of social skills training on behavior disorders among elementry school children. Sid.

[5] Aksoy P., Baran G. (2010). Review of studies aimed at bringing social skills for children in preschool period. Procedia Soc. Behav. Sci..

[6] Gresham F.M., Elliott S.N., Vance M.J., Cook C.R. (2011). Comparability of the social skills rating system to the social skills improvement system: Content and psychometric comparisons across elementary and secondary age levels. Sch. Psychol. Q..

[7] Endedijk H.M., Cillessen A.H., Cox R.F., Bekkering H., Hunnius S. (2015). The role of child characteristics and peer experiences in the development of peer cooperation. Soc. Dev..

[8] Rothbart M.K., Sheese B.E., Rueda M.R., Posner M.I. (2011). Developing mechanisms of self-regulation in early life. Emot. Rev..

[9] Kramer T.J., Caldarella P., Christensen L., Shatzer R.H. (2010). Social and emotional learning in the kindergarten classroom: Evaluation of the strong start curriculum. Early Child. Educ. J..

[10] Moore J.E., Cooper B.R., Domitrovich C.E., Morgan N.R., Cleveland M.J., Shah H., Jacobson L., Greenberg M.T. (2015). The effects of exposure to an enhanced preschool program on the social-emotional functioning of at-risk children. Early Child. Res. Q..

[11] Birch S.H., Ladd G.W. (1997). The teacher-child relationship and children’s early school adjustment. J. Sch. Psychol..

[12] Hosokawa R., Katsura T. (2017). Marital relationship, parenting practices, and social skills development in preschool children. Child. Adolesc. Psychiatry Ment. Health..

[13] Parker J.G., Asher S.R. (1987). Peer relations and later personal adjustment: Are low-accepted children at risk?. Psychol. Bull..

[14] Ziv Y. (2013). Social information processing patterns, social skills, and school readiness in preschool children. J. Exp. Child. Psychol..

[15] Hosokawa R., Katsura T. (2017). A longitudinal study of socioeconomic status, family processes, and child adjustment from preschool until early elementary school: The role of social competence. Child. Adolesc. Psychiatry Ment. Health..

[16] Duncan G.J., Lee K.T., Rosales-Rueda M., Kalil A. (2018). Maternal age and child development. Demography.

[17] Hosokawa R., Katsura T., Shizawa M. (2017). Relations of mother’s sense of coherence and childrearing style with child’s social skills in preschoolers. Child. Adolesc. Psychiatry Ment. Health.

[18] Takahashi Y., Okada K., Hoshino T., Anme T. (2015). Developmental trajectories of social skills during early childhood and links to parenting practices in a Japanese sample. PloS ONE.

[19] Doctoroff G.L., Greer J.A., Arnold D.H. (2006). The relationship between social behavior and emergent literacy among preschool boys and girls. J. Appl. Dev. Psychol..

[20] Abdi B. (2010). Gender differences in social skills, problem behaviours and academic competence of Iranian kindergarten children based on their parent and teacher ratings. Procedia Soc. Behav. Sci..

[21] Shahim S. (2004). Reliability of the social skills rating system for preschool children in Iran. Psychol. Educ..

[22] Gresham F., Elliot S. (1990). Social Skills Rating System.

[23] Persson G.E. (2005). Developmental perspectives on prosocial and aggressive motives in preschoolers’ peer interactions. Int. J. Behav. Dev..

[24] Gouley K.K., Brotman L.M., Huang K.Y., Shrout P.E. (2008). Construct validation of the social competence scale in preschool-age children. Soc. Dev..

[25] Huaqing Qi C., Kaiser A.P. (2003). Behavior problems of preschool children from low-income families: Review of the literature. Topics Early Child. Spec. Educ..

[26] Zhang X., Nurmi J.-E. (2012). Teacher–child relationships and social competence: A two-year longitudinal study of chinese preschoolers. J. Appl. Dev. Psychol..

[27] Koch H., Kastner-Koller U., Deimann P., Kossmeier C., Koitz C., Steiner M. (2011). The development of kindergarten children as evaluated by their kindergarten teachers and mothers. Psychol. Test. Assess. Model..

[28] Gresham F.M., Cook C.R., Vance M.J., Elliott S.N., Kettler R. (2010). Cross-informant agreement for ratings for social skill and problem behavior ratings: An investigation of the social skills improvement system-rating scales. Psychol. Assess..

[29] Oryadi zanjani M., Vahab M., Shahim S., Jafari S. (2012). The relationship of expressive language development and social skills in 4-6-year-old Persian-speaking children. Audiol..

[30] Sheikhzakaryaie N. (2012). Gender differences in social skills of Iranian preschool children. Arch. Sci..

[31] Nourani K. (1999). Social Skills and Adaptive Behavior of Iranian Preschoolers, Teachers’ and Parents’ Ratings. Ph.D. Thesis.

[32] Kaya A., Emine E. (2016). Pre-school period of development. Ann. Nurs. Pract..

[33] Arani A.M., Kakia M.L., Karimi M.V. Assessment in education in Iran. http://www.nwu.ac.za/sites/www.nwu.ac.za/files/files/p-saeduc/New_Folder_1/3_Assessment%20in%20education%20in%20Iran.pdf.

[34] Rich E.C., Shepherd E.J., Nangle D.W. (2008). Validation of the SSRS-T, preschool level as a measure of positive social behavior and conduct problems. Educ. Treat. Children.

[35] Tan T.X., Camras L.A. (2011). Social skills of adopted chinese girls at home and in school: Parent and teacher ratings. Child. Youth Serv. Rev..

[36] Sheikhzakariae N. (2012). Evaluation of Social Skills in Preschool Children in Public Schools in Tehran in 2012. MSc Thesis.

[37] Garmaroudi G., Sadat Vahdani Niya M. (2006). Assessment of students’ social skills. Public Health Q. Payesh.

[38] Razani O., Abdeyazdan Z. (2018). Comparison of social skills of preschool children in single-parent families and two- parent families in doroud city. Nurs. Dev. Health.

[39] Taverna L., Bornstein M.H., Putnick D.L., Axia G. (2011). Adaptive behaviors in young children: A unique cultural comparison in Italy. J. Cross Cult. Psychol..

[40] Dichtelmiller M., Meisels S.J., Plunkett J.W., Bozytnski M.E.A., Claflin C., Mangelsdorf S.C. (1992). The relationship of parental knowledge to the development of extremely low birth weight infants. J. Early Interv..

[41] Mayer S.E. (1997). What Money can’t Buy: Family Income and Children’s Life Chances.

[42] Conger R.D., Conger K.J., Martin M.J. (2010). Socioeconomic status, family processes, and individual development. J. Marriage Fam..

[43] Berry D., O’Connor E. (2010). Behavioral risk, teacher–child relationships, and social skill development across middle childhood: A child-by-environment analysis of change. J. Appl. Dev. Psychol..

[44] Kumari V., Khadi P. (2010). Influence of child’s, parental and familial characteristics on social and personal skills of mentally challenged children. Karnataka J. Agric. Sci..

[45] Sutcliffe A.G., Barnes J., Belsky J., Gardiner J., Melhuish E. (2012). The health and development of children born to older mothers in the United Kingdom: Observational study using longitudinal cohort data. BMJ.

[46] Leigh A., Gong X. (2010). Does maternal age affect children’s test scores?. Aust. Econ. Rev..

[47] Tearne J.E. (2015). Older maternal age and child behavioral and cognitive outcomes: A review of the literature. Fertil. Steril..

[48] Lucas-Thompson R.G., Goldberg W.A., Prause J. (2010). Maternal work early in the lives of children and its distal associations with achievement and behavior problems: A meta-analysis. Psychol. Bull..

[49] Ruhm C.J. (2004). Parental employment and child cognitive development. J. Hum. Resour..

[50] Dadsetan P., Asgary A., Bayat M., Rahimzadeh S. (2011). Parental employment and children cognitive development. J. Educ. Psychol..

[51] Greenstein T.N. (1993). Maternal employment and child behavioral outcomes: A household economics analysis. J. Fam. Issues.

[52] Hamre B.K., Pianta R.C. (2001). Early teacher–child relationships and the trajectory of children’s school outcomes through eighth grade. Child. Dev..

[53] Yoleri S. (2017). Teacher-child relationships in preschool period: The roles of child temperament and language skills. Int. Electron. J. Elementary Educ..

[54] O’Connor E.E., Dearing E., Collins B.A. (2011). Teacher-child relationship and behavior problem trajectories in elementary school. Am. Educ. Res. J..

[55] Jennings J.L., DiPrete T.A. (2010). Teacher effects on social and behavioral skills in early elementary school. Sociol. Educ..

[56] Schanzenbach D.W. Does class size matter?. https://nepc.colorado.edu/publication/does-class-size-matter.

[57] Fredriksson P., Öckert B., Oosterbeek H. (2012). Long-term effects of class size. Q. J. Econ..

[58] Pečjak S., Puklek Levpušček M., Valenčič Zuljan M., Kalin J., Peklaj C. (2009). Students’ social behaviour in relation to their academic achievement in primary and secondary school: Teacher’s perspective. Psihologijske Teme.

[59] Zahra E.D., Nazanin V., Reza E.M., Sima K., Zohreh S. (2014). Implementation of mother-training program to improve parenting in pre-school age children: A randomized-controlled trial. N. Am. J. Med. Sci..

[60] Morawska A., Winter L., Sanders M. (2009). Parenting knowledge and its role in the prediction of dysfunctional parenting and disruptive child behaviour. Child. Care Health Dev..

